# Looking for consistency in an uncertain world: test-retest reliability of neurophysiological and behavioral readouts in autism

**DOI:** 10.1186/s11689-021-09383-0

**Published:** 2021-09-30

**Authors:** Shlomit Beker, John J. Foxe, John Venticinque, Juliana Bates, Elizabeth M. Ridgeway, Roseann C. Schaaf, Sophie Molholm

**Affiliations:** 1 The Cognitive Neurophysiology Laboratory, Department of Pediatrics, Albert Einstein College of Medicine, Bronx, NY USA; 2 Department of Neuroscience, Albert Einstein College of Medicine, Bronx, NY USA; 3 The Cognitive Neurophysiology Laboratory, The Ernest J. Del Monte Institute for Neuroscience, Department of Neuroscience, University of Rochester School of Medicine and Dentistry, Rochester, NY USA; 4 School of Medicine, Albert Einstein College of Medicine, Bronx, NY USA; 5 Department of Occupational Therapy, Jefferson College of Health Professions Faculty, Farber Institute for Neurosciences Thomas Jefferson University Philadelphia, Philadelphia, USA; 6 Department of Psychiatry and Behavioral Sciences, Albert Einstein College of Medicine, Bronx, NY USA

**Keywords:** ASD, ICC, Biomarkers, Inter-trial variability, ERP, EEG

## Abstract

**Background:**

Autism spectrum disorders (ASD) are associated with altered sensory processing and perception. Scalp recordings of electrical brain activity time-locked to sensory events (event-related potentials; ERPs) provide precise information on the time-course of related altered neural activity, and can be used to model the cortical loci of the underlying neural networks. Establishing the test-retest reliability of these sensory brain responses in ASD is critical to their use as biomarkers of neural dysfunction in this population.

**Methods:**

EEG and behavioral data were acquired from 33 children diagnosed with ASD aged 6–9.4 years old, while they performed a child-friendly task at two different time-points, separated by an average of 5.2 months. In two blocked conditions, participants responded to the occurrence of an auditory target that was either preceded or not by repeating visual stimuli. Intraclass correlation coefficients (ICCs) were used to assess test-retest reliability of measures of sensory (auditory and visual) ERPs and performance, for the two experimental conditions. To assess the degree of reliability of the variability of responses within individuals, this analysis was performed on the variance of the measurements, in addition to their means. This yielded a total of 24 measures for which ICCs were calculated.

**Results:**

The data yielded significant *good* ICC values for 10 of the 24 measurements. These spanned across behavioral and ERPs data, experimental conditions, and mean as well as variance measures. Measures of the visual evoked responses accounted for a disproportionately large number of the significant ICCs; follow-up analyses suggested that the contribution of a greater number of trials to the visual compared to the auditory ERP partially accounted for this.

**Conclusions:**

This analysis reveals that sensory ERPs and related behavior can be highly reliable across multiple measurement time-points in ASD. The data further suggest that the inter-trial and inter-participant variability reported in the ASD literature likely represents replicable individual participant neural processing differences. The stability of these neuronal readouts supports their use as biomarkers in clinical and translational studies on ASD. Given the minimum interval between test/retest sessions across our cohort, we also conclude that for the tested age-range of ~ 6 to 9.4 years, these reliability measures are valid for at least a 3-month interval. Limitations related to EEG task demands and study length in the context of a clinical trial are considered.

**Supplementary Information:**

The online version contains supplementary material available at 10.1186/s11689-021-09383-0.

## Background

Autism spectrum disorder (ASD) is defined by social-communication deficits and restricted and repetitive patterns of behavior, and is often accompanied by sensory, motor, perceptual, and cognitive atypicalities. Although well defined by clinical diagnostic criteria and assessed professionally through interviews and clinical observation, ASD is highly heterogeneous, with wide ranging presentation and a variety of etiologies and developmental trajectories [1–3]. As a neurodevelopmental condition, direct measures of brain activity provide for greater understanding of the underlying neuropathology and how this impacts information processing. If robust, replicable, and reliable neurophysiological measures of processing differences in ASD can be developed, these might then have utility in the stratification of individuals at early stages of the condition, to optimize targeted interventions, and as biomarkers for assaying treatment efficacy.

Scalp recordings of electrophysiological brain responses (electroencephalogram: EEG) provide a non-invasive readout of network level neural processing with millisecond temporal resolution. EEG time-locked to stimulus presentation or to behavioral responses, referred to as event-related potentials (ERPs), is used to characterize the time-course of information processing [4, 5], and can also be used to model the cortical loci of the underlying neural networks [6, 7]. EEG/ERPs are thus well-suited to the characterization of when and where cortical information processing might be altered in ASD, and have the potential to provide sensitive assays of treatments that are expected to act on processes with a clear neural signature. Additionally, since EEG/ERPs directly index neural function, they are likely more sensitive to initial treatment effects, given that they can measure site-of-action effects in real-time. This feature is particularly meaningful for clinical trials, which tend to be of relatively short-duration, and would benefit from more sensitive and immediate outcome measures. In contrast, more typical clinical and behavioral assays might be expected to show somewhat delayed treatment-related changes, since neural changes due to intervention would only give rise to changes in behavioral outcomes after sufficient time has passed.

There is an accumulation of support for altered sensory-perceptual processing in ASD, with evidence for differential processing across all the major sensory modalities, including audition [8–12], vision [13–16], somatosensation [17, 18], and multisensory integration systems [19]. However, it bears mentioning that these differences, when present, can often be subtle, and that there has tended to be a high degree of inconsistency across the literature (see [20]). Nonetheless, a promising development is that variance in sensory ERPs has been related to the severity of the clinical phenotype [12, 21], going to their utility as potential biomarkers. A similarly promising development is work showing that both auditory and visual sensory responses can be modulated by training, signifying potential sensitivity to treatment effects [22, 23]. However, sensory ERPs have not yet been submitted to standard assessment of test-retest reliability in ASD, which is surely a minimal requirement in assessing their potential as sensitive biomarkers. Indeed, this seems particularly germane given often inconsistent findings across studies, and suggestions by some research groups of increased inter-trial variability of the sensory evoked response in ASD [2, 24], but see [25, 26].

In the quest for reliable biomarkers to index brain function in ASD, we sought here to measure the test-retest reliability of auditory and visual evoked potentials and related task performance. High-density EEG recordings and behavioral responses were recorded from children with ASD while they engaged in a simple speeded reaction time (RT) task in response to visually cued and non-cued auditory stimuli. Intraclass correlation coefficients (ICCs) [27–29] were calculated to assess the reliability of the sensory evoked responses and behavioral data recorded across two identical experimental sessions that were temporally separated by an average of about 5 months.

## Methods

### Participants

Data from 33 children diagnosed with ASD ranging from 6.1 to 9.4 years of age were included for this analysis (see Table 1 for participant characteristics). These came from a larger dataset (*N* = 94) collected in the context of a clinical trial on the efficacy of different behavioral interventions, and included a subset of the participants from whom we recorded EEG and behavioral data from two sessions, which we refer to as test (pre intervention) and retest (post intervention). Of the 33 participants included in these analyses, two thirds (*n* = 22) were in active treatment groups (applied behavioral analysis (ABA), *N* = 10; sensory integration therapy (SIT), *N* = 12), and one-third in a *treatment as usual* control group (*N* = 11). Data were collapsed across treatment groups due to the relatively small *N*.

**Table 1:** Means ± SD and range of characteristics and cognitive scores of all participants

Sex	Age	Time between tests (months)	Handedness	IQ* (full scale)	IQ (verbal)	IQ (non-verbal)	ADOS severity
29 (M) 4 (F)	7.54 ± 1 [6.1–9.4]	Average: 5.2 ± 2 [2.9–10.4]	Right/Left: 20/13	92.9 ± 16 [58–131]	90.4 ± 20 [56–130]	95.5 ± 16 [64–128]	7.78 ± 1.5 [5–10]

While we had full datasets from test and retest sessions in 40 participants, 7 (17.5%) were excluded from the current analysis because of insufficient data due to artifact contamination in one or both of the recording sessions. See Table 2 for reasons for exclusion and for comparison of the demographics and characteristics of the included versus excluded participants; see also “Discussion” section.

**Table 2:** Reasons for exclusion (A), and statistical comparison of clinical and other characteristics of included and excluded participants (B)

**A) Reasons for exclusion**						
	**Withdrew from the study after 1**^**st**^**session**	**Covid-19 related issues**	**EEG attempted** **but unsuccessful**	**Noisy EEG data**	**Still in intervention**	
***N*= 61**	8 (13.1%)	13 (21.3%)	31 (50.8%)	7 (11.5%)	2 (3.3%)	
**B) Age, Sex and cognitive scores**						
	**Age**	**Sex**	**IQ (Full scale)**	**IQ (Verbal)**	**IQ (Non-verbal)**	**ADOS severity**
**Included in analysis (*N*= 33)**	7.54 ± 1 [6.1–9.4]	29(M) 4 (F)	92.9 ± 16 [58–131]	90.4 ± 20 [56–130]	95.5 ± 16 [64–128]	7.78 ± 1.5 [5–10]
**Excluded from** **analysis (*N*= 61)**	7.46 ± 1 [6–9.5]	50 (M) 11 (F)	83.8 ± 19 [50–124]	75.9 ± 22 [45–128]	92.5 ± 19 [56–143]	8.1 ± 1.7 [3–10]
**Difference (two** **sample*T*test)**	t-stat = 0.1 df = 92 p = 0.87	*χ*^2^stat = 0.56 *p* = 0.45	t-stat = 2.6 df = 92 *p* = 0.01	t-stat = 3 df = 92 *p* = 0.003	t-stat = 0.8 df = 92 *p* = 0.42	t-stat = 0.4 df = 92 *p* = 0.63

The time between test and retest was 5.2 ± 2 months (min: 2.9; max: 10.4). Participants were recruited without regard to sex, race, or ethnicity. IQ quotients for performance (PIQ), verbal (VIQ), and full-scale (FSIQ) intelligence were assessed in all of the participants using the Wechsler Abbreviated Scales of Intelligence (WASI [30];). To be considered for the study, participants had to meet diagnostic criteria for ASD on the basis of the Autism Diagnosis Observation Schedule (ADOS-2) [31], childhood history, and clinical impression of a licensed clinician with extensive experience in the evaluation and diagnosis of children with ASD. The Repetitive Behavior Scale-Revised (RBS-R) [32] questionnaire was collected to obtain continuous measures of ASD characteristics related to insistence on sameness such as ritualistic/sameness behavior, stereotypic behavior, and restricted interests. Participants received modest recompense for their participation (a total of $250 for participation in the treatment study). Exclusionary criteria included epilepsy or premature birth (< 35 weeks). While the majority of participants had non-verbal IQs > 80, a subset (*N* = 6) had lower scores (ranging from 64 to 79, with a mean of 73 and standard deviation of 5.3). All participants passed a screen for normal or corrected-to-normal vision and normal hearing on the day of testing. Parents and/or guardians of all participants provided written informed consent. All procedures were approved by the Institutional Review Board of the Albert Einstein College of Medicine.

### Stimuli and task

**Fig. 1:**
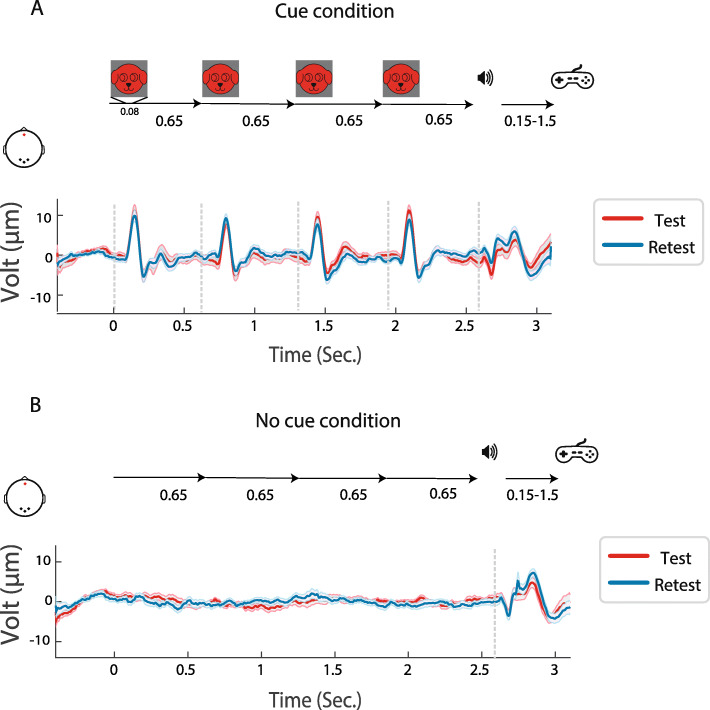
Schematic of experimental paradigm. **A** Top: Cue condition trial. Bottom: Cue condition grand average responses over trial epoch at occipital channels (O1, O2, Oz). **B** Top: No-Cue condition trial. Bottom: grand average of evoked responses for the trial. No-Cue condition grand average responses over trial epoch at occipital channels (O1, O2, Oz)

The paradigm was designed like a computer game, with stimuli that consisted of a cartoon dog face as the visual stimulus, and cartoons of a running, happy, or sleeping dog as feedback for responses to the auditory target that were, respectively, too fast, right on time, or too slow. The visual feedback was accompanied by an uplifting sound (slot machine sound) or a neutral sound (two tones in high-low pitch sequence), for responses falling in/outside the response window, respectively. The visual cue stimuli were presented centrally on a 25” ViewSonic screen (refresh rate: 60 Hz, pixel resolution: 1280 × 1024 × 32) of a Dell computer using Presentation® software (Version 20.0, Neurobehavioral Systems, Inc., Berkeley, CA), and subtending ~ 4.4° of visual angle. The auditory target stimulus was a 1000 Hz tone 80 ms in duration that was delivered at an intensity of 75 dB SPL via a single, from a centrally located loudspeaker (JBL Duet Speaker System, Harman Multimedia) (see Fig. 1 for paradigm schematic and the corresponding grand average ERP responses over the full trial epoch at occipital channels). The task was designed to test the hypothesis that children with ASD do not use temporally predictive information in a typical way [33, 34]. Two conditions were included: For the *Cue* condition, participants were presented with a sequence of 4 visual isochronous stimuli for a duration of 80 ms each presented at a Stimulus Onset Asynchrony (SOA) of 650 ms, followed by an 80 ms auditory stimulus, presented 650 ms after the onset of the last visual cue. For the *No-Cue* control condition, the auditory stimulus was not preceded by a sequence of visual cue stimuli. Both conditions included 15% catch trials on which the auditory target was not presented. Each target appeared 2600 ms after the beginning of the trial, during which participants were focused on the screen. In all other respects, the paradigm, including the timing of the stimuli, was identical between the Cue and No-Cue conditions. Cue and No-Cue conditions were presented in blocks, with 25 trials per block, and a total of 20 blocks (10 Cue; 10 No-Cue). Each block lasted 3.5 min, and the order of blocks within the experiment for a given participant was randomly generated prior to each experimental session. Participants were encouraged to take short breaks between blocks as needed. The entire experimental session lasted around 3 h, and, in addition to data acquisition, included cap application, frequent short breaks, lunch, and cap removal. Participants were seated at a fixed distance of 65 cm from the screen and responded with their preferred hand. In all trials, they were instructed to press a button on a response pad (Logitech© Wingman Precision Gamepad) as soon as they heard the auditory tone. Responses occurring between 150 and 1500 ms after the auditory target stimulus were considered valid, and positive feedback of a cartoon dog image and an uplifting sound was provided. If the response was outside this time window, a running dog cartoon with a sad sound was presented to indicate that the response was too fast, and a sitting dog image with the sad sound was presented to indicate that the response was too slow. Frequent breaks were given as needed to ensure maximal task concentration. Here, we focus on the behavioral and sensory evoked responses to evaluate their reliability between two data recording sessions separated by a minimum of 10 weeks (2.5 months). A case-control study testing the hypothesis that children with ASD do not use temporally predictive information in a typical way is presented in a separate report [34].

#### Data acquisition

Response times were recorded with Presentation® software. EEG recordings were collected from 70 active channels (10–20 system; 64 scalp channels and 6 external electrodes: 2 upper mastoids; 2 lower mastoids; 2 vertical EOG) at a digitization rate of 512 Hz, using Active Two (BioSemi™, Amsterdam, The Netherlands) with an anti-aliasing filter (− 3dB at 3.6 kHz). Analog triggers indicating the timing of stimulus onsets and button presses were sent to the acquisition PC via Presentation® and stored digitally at a sampling rate of 512 Hz, in a separate channel of the EEG data file.

### Eye tracking

To ensure that participants adhered to our instruction to fixate centrally, we monitored eye-position throughout the experiment using the Eyelink100® eye-tracking system (sampling rate: 1000 Hz) and with video monitoring. If the experimenter noticed through video monitoring that the child was looking away, or if the eye tracking system indicated that gaze moved away from the screen, the experimenter reminded the participant to look at the centrally placed fixation cross.

### Data processing and analysis

Data were processed and analyzed using custom MATLAB® scripts (MATLAB r2017a, MathWorks, Natick, MA), and the FieldTrip toolbox [35]. A minimum number of 50 EEG trials per analysis was set as a criterion for a participant to be included in the analysis; however, most participants had more than 100 trials in each condition and session (e.g., for auditory trials, Cue condition; test: mean ± standard deviation (SD): 208 ± 86; retest: 209 ± 95). Due to occasionally extreme RT in some of the participants, the tails of the RT distributions of each participant (2.5% at each end) were excluded from further analysis.

Measurements that were used in the ICC analysis were calculated as follows:

1. Behavior

RT and sensitivity indexed by d-Prime (*d*′) were calculated from the behavioral data [36, 37]. Hits were defined as responses that occurred between 150 and 1500 ms following the auditory tone. Proportion of hits was defined as the ratio between the number of hits and the number of all targets presented to the participant. A false alarm was defined as a response to a catch trial (i.e., pushing the button even though no auditory target stimulus occurred). The proportion of false alarms was defined as the ratio between the number of false alarms and the number of all catch trials presented to the participant.

Kolmogorov-Smirnov test of normality of distribution showed a normal RT distribution. Hence, both means and standard deviations (SDs) per participant were used to assess ICC for mean and inter-trial variability (ITV) metrics of RT, respectively. *d*′ was calculated for each participant as the difference between the proportions of the hits and false alarms of the values, after they were transformed to z-scores: *d*^′^ = *Z*(*p*(Hit)) − *Z*(*p*(False Alarm)). The ICC was calculated for RT means and SDs, and for *d*′, for both Cue and No-Cue conditions.

2. EEG data processing

Continuous EEG data were down-sampled to 256 Hz, band-pass filtered between 0.1 and 55 Hz using Butterworth Infinite Impulse Response (IIR) windowing with filter order of 5, and then epoched as specified below. Epochs were demeaned to normalize for DC shifts, and baseline-corrected using the 100 ms time window prior to stimulus onset. After epoching, a two-stage automatic artifact rejection was applied at the single trial level. First, channels that varied from the mean voltage across all channels and from the auto-covariance by 1 standard deviation were classified as bad. A maximum of six bad channels was set as an inclusion criterion for trials to be analyzed. For these trials, channels were interpolated using the nearest neighbor spline [38, 39]. Second, a criterion of ± 120 μV was applied. Electrodes that exceeded this criterion were considered bad. The EEG components were calculated as follows:

i. Visual evoked response (VEP): To derive the VEP, epochs of 200 ms before and 850 ms after visual stimulus presentation were generated and baselined to the 100 ms pre stimulus onset, and then averaged across trials separately for each participant and recording session. Data were referenced to a midline frontal channel (AFz) to optimize visualization and measurement of the VEP over occipital scalp. In accordance with the literature (e.g., [5]) and confirmed by visual inspection of the data, amplitude values from occipital channels (O1, O2, Oz), at the maxima/minima of each participant’s visual P1 (80–160 ms), N1 (150–210 ms), and P2 (300–400 ms) response were taken for subsequent statistical analyses. Both means and SDs per participant were used to assess ICC for mean and ITV metrics of the VEP, respectively, for a total of 6 measures across the three visual components.
ii. Auditory evoked response (AEP): To derive the AEP, epochs of 300 ms before and 850 ms after auditory stimulus presentation were generated and baselined to the 100 ms pre stimulus onset, and then averaged across trials separately for each participant and recording session, and in both Cue and No Cue conditions. Data were referenced to a channel near the left mastoid (TP7) to optimize visualization and measurement of the AEP over fronto-central scalp. In accordance with the literature (e.g., [40, 41] and confirmed by visual inspection of the data, amplitude values from fronto-central channels (FC1, FC2, FCz), at the maxima/minima of each participant’s auditory P1 (30–80 ms), N1 (80–150 ms), and P2 (160–240 ms) components were taken for subsequent statistical analyses.

Each of the 6 behavioral measurements and the 6 VEP and the 12 AEP components elaborated above were calculated for each participant for each of the two sessions. Two datasets, one VEP and one AEP, each from a different subject, did not meet criteria for inclusion and were excluded from the analysis. Hence, ICC of both VEP and AEP components was calculated on 32 of the 33 participants.

### Test-retest analysis

Our analyses focused on assessing the consistency of behavioral and electrophysiological responses across two recording sessions. To do this, we performed intraclass correlation coefficient (ICC) analyses using a one-way mixed effect model with absolute agreement and multiple observations [27, 29, 42, 43], according to the formula:

$$ \mathrm{ICC}\left(1,k\right)=\frac{{\mathrm{MS}}_R-{\mathrm{MS}}_w}{{\mathrm{MS}}_R} $$

MS_R_ = mean square for rows (variance between participants); MS_W_ = mean square for residual sources of variance; *k* = number of raters (or measurements, in this case *k* = 1).

Separate ICCs were calculated for test-retest pairs for each of the 24 measurements. ICC was computed with the Intraclass Correlation Coefficient package:

https://www.mathworks.com/matlabcentral/fileexchange/22099-intraclass-correlation-coefficient-icc, MATLAB Central File Exchange (Arash Salarian, 2020). To correct for multiple comparisons, Bonferroni correction [44] was applied to the ICC values.

Testing for association between test-retest similarity and participant cognitive variables: First, a test-retest similarity index (SI) was calculated for each individual, indicating the degree of similarity between the test and retest across all measurements. SI was calculated on all test-retest pairs as following:

$$ Y={Z}_{score}\left({X}_1\dots {X}_n\right),\kern1em {SI}_n=1-\mathit{\operatorname{var}}\left({\sum}_{k=i}^m {\;{Y}_{n} {/}{m}}\right)$$

*X*= Measurement (ERP or behavior), *n* = number of participants, *m* = number of measurements (ERP and behavior).

For purely descriptive purposes, Pearson linear correlation coefficients were calculated for the pairs of observations (test/retest) for each of the behavioral and ERP parameters. While such correlations do not account for absolute agreement between the values across sessions as does ICC, they allow for visualization of the relationship between test and retest measures. Pearson correlation was computed as: $\rho =\frac{\mathit{\operatorname{cov}}\left(X,Y\right)}{\sigma X\bullet \sigma Y}$. Cov = covariance of test and retest; *σX* and *σY* are the SD of test and retest, respectively. To control for false discovery rate (FDR), Bonferroni correction [44] was applied on all *p* values of all correlations.

To measure for possible associations between the SI and participant cognitive variables, Pearson correlation coefficients were calculated between SI, PIQ, VIQ, RBSR, and ADOS, in the form of a correlation matrix. Results were then corrected for multiple comparisons [44].

Finally, to test for the possibility that test-retest reliability found for the participants was linked to the participant age, number of trials, or to the time that had passed between the sessions, which varied quite widely between 2.9 and 10.4 months, we measured the correlation between the participants’ similarity index and the each of those variables: age, the number of visual trials included per participant, and the test-retest time interval.

### The influence of number of trials on signal-to-noise-ratio and ICC

In addition to the above analyses, we considered the signal-to-noise-ratio (SNR) of the auditory and visual ERPs, reasoning that the differing number of trials that went into the auditory and visual ERPs might have influenced SNR. In turn, we considered how decreasing the number of trials that went into the visual measures would influence SNR and ICC values. We first measured SNR [45] for visual and auditory evoked responses, separately. We used the pre-stimulus period of − 100 to 0 ms as an estimate of background noise, and the corresponding visual P1 and auditory P2 values (as described above) as an estimate of the signal. Signal was divided by noise and converted to decibels in order to be scale-invariant. The resulting SNRs were compared between the two modalities using an unpaired *t* test.

To assess how reducing the number of trials that went into the visual ERP influenced ICC and SNR values, we calculated these using 25% of visual trials, to be comparable to the number of trails that went into the AEPs, and 12.5% of visual trials, which yielded mean ± SD = 117 ± 39 trials (min: 28 max: 192). Trials were selected randomly from the whole set to achieve the subsets, and this was done 5 times each for the 25% and 12.5% subsets, and ICC and SNR calculated for each. The reported ICC and SNR values are based on the average of these 5 ICC and SNR values.

## Results

**Fig. 2:**
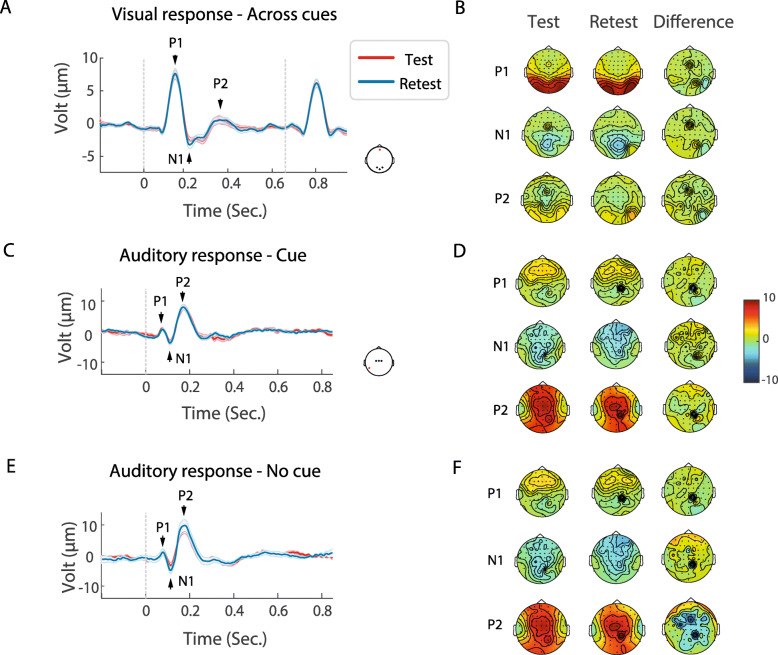
ERPs: visual evoked potentials (VEP) and auditory evoked potentials (AEP) in the two test sessions. **A** VEP (averaged over channels O1, O2, and Oz) collapsed across all visual evoked responses, in test (red) and retest (blue). **B** Topography maps for the VEP P1 (1st row), N1 (2nd row), P2 (3rd row) components shown in (**A**) for (from right to left): test, retest, and the difference between them. **C** AEP (averaged over channels FC1, FC2, FCz) for test and retest in the Cue conditions. **D** Topography maps for the AEP P1, N1, P2 components in Cue condition, for test, retest, and the difference between them. **E** AEP for test and retest in the No-Cue condition. Same as in (**D**), for No-Cue condition

**Fig. 3:**
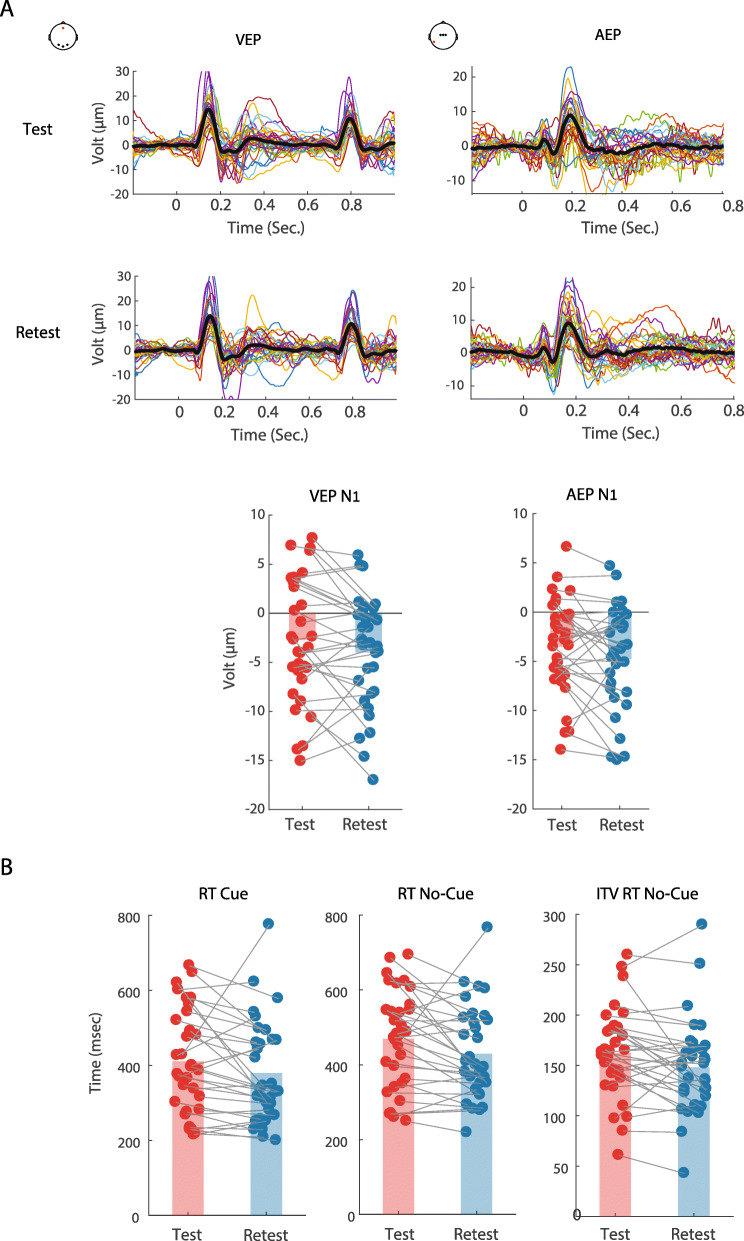
Individual-level ERPs and reaction times (RT) for test and retest. **A** Top, ERPs showing VEP (left) and AEP (right) for test and retest, for all participants (each colored line represent an evoked response of an individual participant). Black: grand average for each session. Bottom, illustration of measurement consistency for the ERP data that showed the highest ICC scores: amplitudes of the visual N1 (left) and auditory N1 (right) at test and retest. **B** Illustration of measurement consistency for the behavioral data that showed the highest ICC scores: reaction times (RT) for the Cue (left) and No-Cue (middle) conditions, and inter-trial variability (ITV) of RT for No-Cue (right), at test and retest

The auditory and visual sensory evoked responses at test and retest are illustrated in grand average VEP and AEP waveforms and topographic maps in Fig. 2, and the individual participant VEP, AEP and behavioral responses in Fig. 3. A striking similarity between the group mean responses can be observed in Fig. 2, whereas at the individual participant level, in Fig. 3, some variance is apparent. ICC analyses were performed to formally assess the consistency of responses at the individual participant level between test and retest.

### Intraclass correlation coefficient

**Fig. 4:**
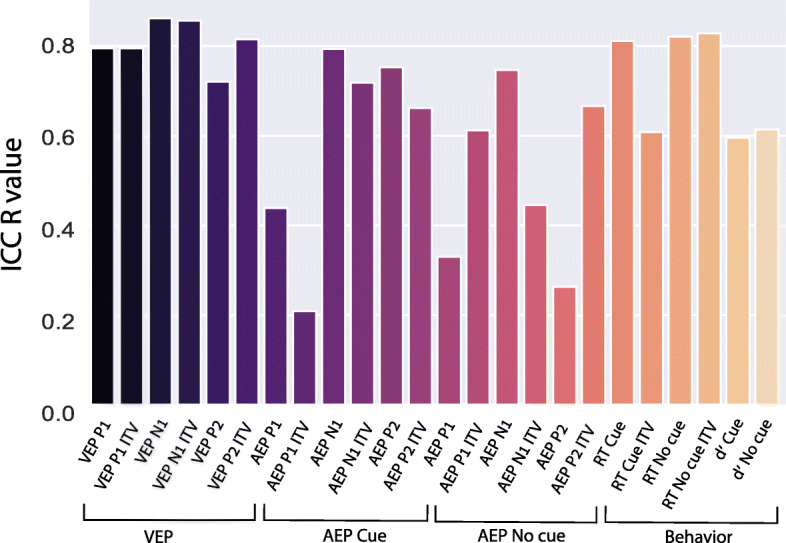
ICC values, grouped by measurement type: VEP, AEP (Cue and No-Cue), and Behavior

Twenty-four measures of behavioral and EEG data (see methods) were submitted to intraclass correlation coefficients (ICC) analysis. ICC *R* values are presented in Fig. 4. ICC *R* values, *p* values and lower and upper bounds of the 95% confidence interval, calculated separately for each measurement, are presented in Table 3.

**Table 3:** *R* values, *p* values, and upper bounds (UB) and lower bounds (LB) of the 95% confidence interval for test-retest, for each of the measurements, ranked from the highest ICC value to the lowest. In italics: significant measurements after correction for multiple comparisons

Measure	***R*** value (ICC)	***P*** value	UB	LB
**ICC** > **0**.**75**				
*VEP N1*	*0.8615*	*1.2359* × *10*^−*7*^	0.9321	0.7185
*VEP N1 ITV*	*0.856*	*1.9969* × *10*^−*7*^	0.9294	0.7072
*RT NC ITV*	*0.8282*	*1.1297* × *10*^−*6*^	0.9149	0.6548
*RT NC*	*0.8201*	*1.9527* × *10*^−*6*^	0.9108	0.6385
*VEP P2 ITV*	*0.8148*	*3.8236* × *10*^−*6*^	0.9093	0.6236
*RT Cue*	*0.811*	*3.4601* × *10*^−*6*^	0.9063	0.6203
*VEP P1*	*0.7946*	*2.4445* × *10*^−*5*^	0.8993	0.5825
*VEP P1 ITV*	*0.7944*	*1.2216* × *10*^−*5*^	0.8992	0.582
*AEP N1 Cue*	*0.7926*	*1.2074* × *10*^−*5*^	0.8984	0.5785
*AEP P2 Cue*	*0.7518*	*8.7397* × *10*^−*5*^	0.8784	0.4955
**0.5 < ICC < 0.75**				
*AEP N1 NC*	*0.7462*	*1.0912* × *10*^−*4*^	*0.8757*	*0.4842*
*VEP P2*	*0.7203*	*2.8088* × *10*^−*4*^	*0.8629*	*0.4314*
*AEP N1 Cue ITV*	*0.7181*	*3.0223* × *10*^−*4*^	*0.8619*	*0.427*
*AEP P2 NC ITV*	*0.6657*	*0.0014*	*0.8362*	*0.3204*
*AEP P2 Cue ITV*	*0.6616*	*0.0016*	*0.8342*	*0.3121*
D′ No-Cue	0.6136	0.004	0.8084	0.2234
AEP P1 NC ITV	0.6114	0.0048	0.8096	0.2102
RT Cue ITV	0.6082	0.0045	0.8058	0.2125
D′ cue	0.595	0.0058	0.7992	0.186
**ICC < 0.5**				
AEP N1 NC ITV	0.4451	0.0513	0.7281	-0.128
AEP P1 Cue	0.4387	0.0547	0.725	-0.1408
AEP P1 NC	0.3302	0.1324	0.6718	-0.3615
AEP P2 NC	0.2632	0.1973	0.639	-0.4976
AEP P1 Cue ITV	0.2088	0.2567	0.6123	-0.6081

Visual evoked response (VEP), inter-trial variability (ITV), reaction time (RT), no cue (NC), auditory evoked response (AEP)

For ICC analysis, the higher the *R* value is, the stronger the agreement between the two sessions. Per convention, values below 0.50 are generally considered to have a *poor* level of reliability, values from 0.50 to 0.75 to be of *moderate* reliability, values from 0.75 to 0.90 to have *good* reliability, and, when higher than 0.90 they are considered to have *excellent* reliability [46]. Note that other categorization criteria for ICC values have been suggested. For example, by Fleiss’s [47] scheme, in which *R* > 0.75 is categorized as *excellent*, the top 10 measures in Table 3 would be considered *excellent*, rather than *good*. After applying Bonferroni correcting for multiple comparison, the following 10 measures with ICC > 0.75 remained significant: RT Cue, RT No-Cue, RT No-Cue ITV, VEP P1, VEP P1 ITV, VEP N1, VEP N1 ITV, VEP P2 ITV, AEP N1 Cue, and AEP P2 Cue. In the Pearson correlations that we performed for descriptive purposes, significant correlations for all measurements but AEP P1 No Cue, AEP P2 No Cue, and AEP P1 ITV were found following correction for multiple comparisons. Pearson correlations for the test-retest pairs are presented in Supplementary Fig. 1.

### ICC and signal-to-noise ratio

Comparison of signal-to-noise-ratio (SNR) between the VEP and AEP show significantly reduced SNR for the AEP compared with VEP (see Table 4).

**Table 4:** Mean ± SD for SNR in visual P1 and auditory P2

	Visual (P1)	Auditory (P2)	***t*** test
**SNR**	**55** ± **18**	**23** ± **29**	***t*= 5.5; df = 61;*p*< 0.001**
N trials	922 ± 328	208 ± 86	

**Fig. 5:**
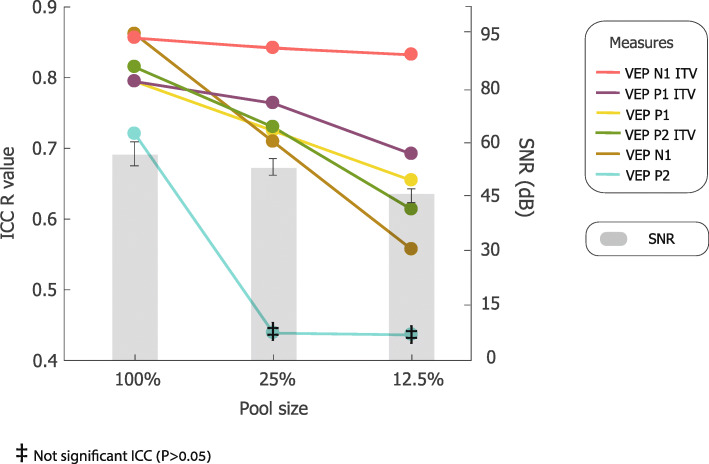
ICC values (color lines; left y axis) and SNR (gray bars; right y axis) as a function of number of visual trials used in the calculation

ICC and SNR values for two subsets of the VEP trials, 25% and 12.5% of the general pool, were calculated to assess how number of trials influenced ICC and SNR (see Fig. 5). Each reduction of the pool size resulted in lower ICC values for all 6 measures tested. The mean ICC values for VEP components for the 100%, 25% and 12.5% sets of trials are 0.81, 0.70, and 0.63, respectively. This reduction is in accordance with the SNR values, which are different between the 25% (SNR mean ± SEM = 50.4 ± 2.2) 12.5% (45.1 ± 2), and 100% sets (55.6 ± 3; ANOVA *F* = 4.28; df = 95; *p* = 0.016). Tukey-Kramer post-hoc test revealed a significantly lower SNR value for the 12.5% compared to the 100% set (*p* < 0.01).

### Similarity index and clinical measures

Similarity index (SI) was generated for each participant (see “Methods” section) and tested for correlation with ADOS severity scores, PIQ, VIQ, and RBSR in a correlation matrix (Supplementary Figure 2). None of these correlations survived Bonferroni correction [44]. Finally, no correlation was found between SI and age (Rho = − 0.11; *p* = 0.54), SI and number of visual trials per participant (rho = − 0.21; *p* = 0.24), or SI and the between-sessions time interval (rho = 0.006; *p* = 0.97).

## Discussion

In autism research, several factors bring into question the possibility that brain measurements can serve as reliable markers of neurocognitive function. Basic findings on sensory processing from recordings of electrophysiological brain activity often differ across laboratories; and there is some evidence of higher inter-participant [48–50] and inter-trial ([51–53], but see [25, 26]) variability within such recordings compared to control groups. This raises the possibility that such measurements may simply be too noisy to serve as reliable readouts of brain function in ASD. Alternatively, differences in findings between laboratories may result from factors that do not have direct implications for the reliability of the scalp recorded electrical brain response, such as differences in stimuli, task, EEG recording setup and analysis pipeline, ascertainment bias, and clinical cohort. What is more, inter-participant variability may reflect a feature of the heterogeneity of the disorder rather than random noise. Surprisingly few studies to date have sought to test the stability of these responses when participants, recording equipment, analytic approach, and stimulation parameters are held constant, which is particularly critical to establish if a biomarker is to be used as an outcome measure in a clinical trial, or as a reliable indicator of neural and neurocognitive dysfunction [54, 55]. Only two previous studies, as far as we are aware, examined the reliability of such measures in ASD across two recording sessions. Levin and colleagues [56] collected 5 min of resting state EEG from children with and without ASD at two intervals separated by ~ 6 days, and found *good* reliability of the center frequency and amplitude of the largest alpha-band peak. Cremone-Caira and colleagues [57] found *moderate* to *good* reliability of the executive function related frontal-N2 response elicited during go/nogo and flanker tasks in children with ASD across two time points separated by ~ 3 months.

Here, we add to this emerging literature with the finding that in children with ASD, auditory and visual ERPs, as well as reaction-times collected in an accompanying target detection task, show *good* test-retest reliability. We found statistically significant test-retest reliability, as measured by the intraclass correlation coefficient, for a full 15 of the 24 electrophysiological and behavioral measurements submitted to analysis, with significant ICC values ranging from 0.65 to 0.86 (representing *moderate* to *good* ICC values [46, 47]). Interestingly, these high ICC values were found not only for mean responses but also for the inter-trial variability (ITV) of these responses. Significance was found across data category (ERP and behavior), sensory domain (VEP and AEP), ERP component (P1, N1, and P2), experimental condition (Cue and No-Cue), and response metric (mean and ITV). Among the 9 measurements for which significant ICCs were not found, 6 were from the AEP (representing 50% of the AEP derived measures). In notable contrast, ICC was significant for all measurements of the VEP. This difference may be partly accounted for by a higher SNR for the visual ERP. However, when number of trials was more-or-less equated between auditory and visual ERPs, SNR was still substantially higher for the visual compared to the auditory dataset (*t* = 5.05; df = 61; *p* < 0.01).

A follow up analysis on number of visual trails and their relationship to SNR and ICC values showed that while reducing the number of trials only had a moderate, although significant, effect on SNR, ICC values for the ERPs were substantially impacted, dropping dramatically in some cases (see Fig. 5). This suggests that amount of data collected is an important consideration when using EEG as an outcome measure in clinical trials. Notably, however, in the vast majority of cases when considering only 25% of the trails (mean ± SD = 234 ± 79), ICC values were 0.7 or greater and were significant at *p* < 0.05. In contrast, reducing the dataset size further to ~ *N* = 117 resulted in a major reduction of ICC values for most of the visual measures. It is noteworthy that some of the ICC values for the behavioral measures, which were calculated on the same number of trials as the AEP, were among the highest, indicating that the behavioral data required fewer trials to stabilize than the EEG data.

Given the minimum interval between test/retest sessions across our cohort, we conclude that for the tested age-range of ~ 6 to 9.4 years, these reliability measures are valid for at least a 3-month interval. These results add to a still small but growing body of evidence for *good* test-retest reliability of the EEG/ERP response in ASD [56, 57], and extends these findings to the early cortical sensory components as well as related RT data, and for a longer test-retest interval than previously shown. However, we also show that dataset size is an important variable for test-retest reliability, and that, at least for the current paradigm, a relatively large dataset is required for *good* test-retest reliability for visual ERPs. Clearly, many variables will influence the amount of data required for a reliable signal; further, one must consider the trade-off between extent of reliability of the signal and feasibility of test duration for a given population and circumstances.

Atypicalities in sensory evoked neural responses and behavioral performance have been widely reported in ASD, including altered responses to visual [2, 49, 58], auditory [59], and somatosensory stimuli [60, 61]. Moreover, in some studies, higher inter-participant [48–50] and within-participant inter-trial [51–53] variability of brain responses to sensory stimuli has been shown. This is in line with higher inter-trial behavioral variability that was observed for individuals with ASD, measuring reaction times to executive function [62] and tactile judgment tasks [63], as well as rhythmic tapping tasks [64]. The higher variability between trials and between individuals with ASD has, in turn, been interpreted in the context of neuronal processing being “noisy” or “unreliable” (e.g., [49, 50, 52, 65–67], but see [68, 69] and [25, 26] for reports of lower, or typical levels of noise in ASD, respectively). According to this view, high levels of endogenous neural noise in ASD render neural signals unreliable [53, 70]. Arguing against a pure noise account, here we see a stable pattern of both mean activity and ITV over time. The current data suggest that such variance likely represents replicable neural processing differences at the individual participant level in the clinical group, rather than noise. Hypo- and/or hypersensitivity of synaptic activity, for example, could lead to a higher than typical range of neuronal responses to a given stimulus [71]. A possible result would be an increased range of neural activity across large-scale neural networks that is nevertheless stable over time [25]. At the same time, given the consistency of individual responses within our clinical group, the inter-participant variability that has been observed in ASD [48, 50] is likely to reflect that ASD has a variety of etiologies and developmental routes [1], that in turn lead to heterogeneous neural and behavioral phenotypes.

A number of notable recent reviews have focused on the promise of EEG-based biomarkers of IDDs, and discussed the requirements and challenges therein [54, 72–74]. Biomarkers have the potential to serve many purposes including assessment of risk, diagnosis, disease progression, intervention response, and mechanism of disease. Validity and reliability of the potential biomarker are critical to establish. Here, we find *good* reliability of sensory evoked responses to simple auditory and visual stimuli using an active paradigm suitable for children. Since auditory and visual sensory ERPs have been shown to differ in ASD, the additional finding that they can be reliably measured and show stability within individuals over time opens the door to their further development as biomarkers. Next steps will be to establish if these measures are equally reliable in the absence of a task and how they are affected by state (e.g., drowsy versus alert), to determine if they can be applied in more severely affected individuals [75–77]. Given the simplicity of the paradigm and stimuli, such biomarkers could also be suitable for translational studies in non-human models of ASD (see discussion by [74]).

We should note that full datasets were collected for fewer than half of the potential cohort. Consideration of the reasons for this, and the implications for EEG biomarker use in clinical studies, is worthwhile. The parent study, a clinical trial, required a minimum of ~ 40 lab visits. During these visits, clinical assessments were performed, collection of primary outcome measures was made at three time-points, and therapy sessions occurred. Due to the already significant demands of the parent study, EEG recordings were not prioritized since they did not provide a primary outcome measure. In this context, about half of the participants that completed the parent study did not yield full EEG datasets (*N* = 31): 32% did not perform the task correctly or at all and so EEG data collection was terminated, 29% would not wear the cap, 16% refused to continue the EEG experiment partway into data collection, 13% did not sit still enough to acquire *good* EEG data and so data collection was terminated, and for 10% either no attempt at EEG data collection was made or hairstyle prevented adequate cap application. Participant characteristics for included and excluded participants are presented in Table 2. Most notably, verbal IQ and full scale IQ were significantly higher for the included group. Otherwise, participant demographics and characteristics appeared to be highly similar.

This brings to light possible challenges for EEG-data collection in clinical trials in pediatric populations with neurodevelopmental disorders, especially when using a paradigm in which participants perform an active task. Use of a passive auditory or somatosensory paradigm while watching a movie with the sound off, an approach that we often take with lower functioning individuals, would have obviated issues of task compliance, and may also have reduced boredom and hyperactivity. Indeed, *good*-to-*excellent* test-retest reliability was shown in a group of Fragile-X participants with substantially lower IQ than in the current study, for AEPs recorded using a passive oddball stimulation paradigm and a 32-channel EEG montage [78]. Nevertheless, the impetus is on us, as researchers with the goal of developing EEG biomarkers that can be used as outcome measures in clinical trials, to develop approaches for pediatric clinical populations that allow EEG data collection in a wider range of circumstances. Low montage EEG recordings (e.g., [79, 80]), using wireless technology, and embedding of stimuli in movies or highly engaging video games or stories [81] are just some adaptations that may increase participant compliance. These are not yet commonly used, if at all. The validity and reliability of EEG measurements under such conditions are not known, and will have to be established each time significant methodological changes are introduced.

We note that when EEG data collection is primary to the study that the participant has been recruited for and therefore is prioritized, we typically have high levels of compliance of at least 85%. In our EEG studies in high functioning clinical populations, we achieve at least this rate of compliance even when we use paradigms that involve relatively complex tasks. What is more, we have similar compliance rates in our EEG studies in lower functioning populations such as Rett Syndrome [75–77] and Batton Disease, where we use passive paradigms that do not require task performance, and in studies on individuals with severe neuropsychiatric conditions [82, 83].

ICC is a strong metric of the reliability of a response for a given group, but it does not provide individual scores that can be used to assess how test-retest similarity may vary as a function of another variable such as the time interval between measures. We therefore generated a composite measure for each individual, the similarity index (SI), which is simply the mean of the variance between test and retest values across all of the z-scored ERP and behavioral measures. The SI may be considered a composite measure of the stability within an individual of the neuronal/behavioral readouts, evoked by a given task. The more similar the test-retest readouts of a process are, the more stable and less variable the neuronal activity that underlies this process. The SI was used to test if the reliability of the measurements systematically varied with three parameters that were variable between the participants: age, number of visual trials, and the time interval between test and retest (the latter of which arose due to uncontrolled factors such as appointments being rescheduled). There was no evidence for a relationship between SI and any of these parameters. We additionally tested for possible covariance of SI scores with participant traits, as represented by cognitive/clinical variables, but found no significant correlation between SI and RBSR, IQ or autism severity scores. Future work will be required to establish the validity of such a composite SI.

### Study limitations

A potential limitation of the current analysis is that the data were collected in the context of a treatment study. Importantly however, with regard to hypotheses for the parent study, there was no expectation that the treatments would influence the basic auditory and visual sensory responses or RTs that we focused on here, and for which we found *good* reliability.

Lastly, while our study finds strong consistency of neuronal and behavioral measurements in children with ASD, it does not include similar data from an age-matched typically developing (TD) control group, and thus we cannot draw conclusions regarding whether reliability differs from a healthy control group and how. However, this does not detract from evidence for remarkably *good* consistency of the responses between two recording sessions in children with ASD and all its implications, which is a critical feature for a treatment biomarker.

## Conclusions

The present data show that sensory ERPs and related behavior can be highly reliable across multiple measurement time-points in ASD. The data further suggest that the inter-trial and inter-participant variability reported in the ASD literature likely often represents replicable individual participant neural processing differences. The stability of these neuronal readouts supports their use as biomarkers in clinical and translational studies on ASD, although manipulation of the size of the visual dataset reveals that better test-retest reliability is found when using larger amounts of data.

## Supplementary Information

**Additional file 1: Supplementary Figure 1.** Pearson correlations for test-retest pairs. Results are shown for all behavioral (red) and evoked sensory (blue) ERP measures used in the study. Rho and p values for show significant correlations for all but high-order EEG measures. NC: No-Cue.

**Additional file 2: Supplementary Figure 2.** Correlation matrix of clinical scores and Similarity Index (SI). Gray scale colors code for Pearson rho. Uncorrected P values are given for each correlation.

## Data Availability

The authors will make the full de-identified dataset with appropriate notation and any related analysis code available in a public repository (Figshare) and include digital object identifiers within the final text of the paper, so that any interested party can access them.

## Abbreviations

ASD: Autism spectrum disorder
EEG: Electroencephalogram
ERP: Evoked response potentials
ICC: Intraclass correlation coefficients
RT: Reaction time
SD: Standard deviation
df: Degrees of freedom
VEP: Visual evoked response
AEP: Auditory evoked response
ITV: Inter-trial variability
NC: No-Cue
