# Supplementary figures and images for: Looking for consistency in an uncertain world: test-retest reliability of neurophysiological and behavioral readouts in autism
Source: J Neurodev Disord. 2021 Sep 30;13:43. doi: 10.1186/s11689-021-09383-0 (PMC8483424; doi:10.1186/s11689-021-09383-0)

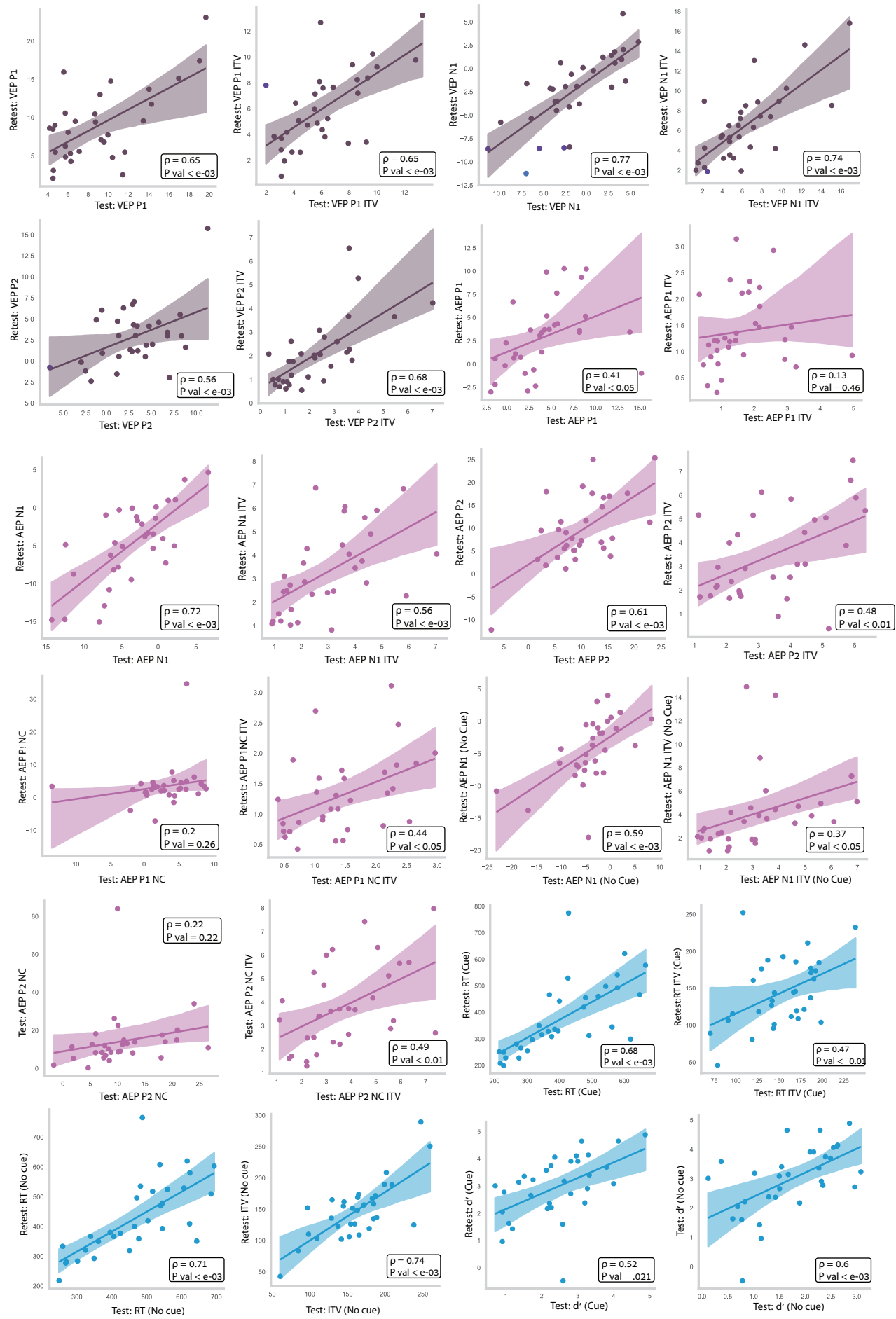

Supplement: Supplementary file 1 — Additional file 1: Supplementary Figure 1. Pearson correlations for test-retest pairs. Results are shown for all behavioral (red) and evoked sensory (blue) ERP measures used in the study. Rho and p values for show significant correlations for all but high-order EEG measures. NC: No-Cue. [file 11689_2021_9383_MOESM1_ESM.pdf]

# clinical scores correlation matrix

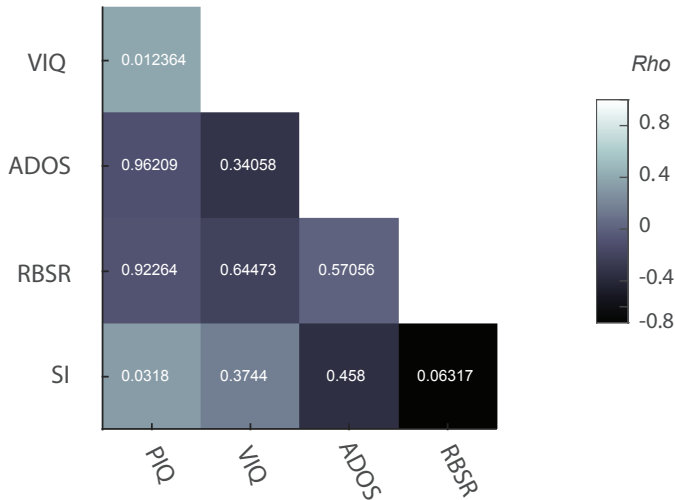

Supplement: Supplementary file 2 — Additional file 2: Supplementary Figure 2. Correlation matrix of clinical scores and Similarity Index (SI). Gray scale colors code for Pearson rho. Uncorrected P values are given for each correlation. [file 11689_2021_9383_MOESM2_ESM.pdf]
